# Sensorineural hearing loss: a new manifestation of neonatal lupus erythematosus?

**DOI:** 10.1186/1546-0096-12-S1-P332

**Published:** 2014-09-17

**Authors:** Ilaria Pagnini, Gabriele Simonini, Teresa Giani, Edoardo Marrani, Valeria Paganelli, Rolando Cimaz

**Affiliations:** 1 Aou Meyer- Rheumatology Dpt, Florence, Italy

## Introduction

Neonatal lupus erythematosus (NLE) is a rare acquired autoimmune disease, characterized by the transplacental passage of maternal antibodies (anti-SSA/Ro and/or anti-SSB/La) into the fetal circulation system, inducing clinical manifestation in the neonate. The most serious clinical manifestation of NLE is a nonreversible complete congenital heart block (CHB), and other common manifestation included skin rash, hepatobiliary and hematologic abnormalities, which are usually reversible.

## Objectives

Central nervous system involvement is not uncommon, but up to now there have been no reports of inner ear involvement in NLE.

## Methods

We describe the clinical case of a 13-year-old Caucasian boy, born from mother with Sjögren syndrome and anti-SSA/Ro antibodies, who developed a complete congenital heart block (CHB), treated with pacemaker implantation at birth. No other clinical manifestations were present in the perinatal period, and anti-SSA/Ro antibodies positivity disappeared in the first 6 months of life.

## Results

When 12-year-old he developed nausea, nystagmus, dizziness, tinnitus, vertigo, and hearing loss, so he was admitted in a community hospital, where laboratory test revealed leucocytosis, with normal CRP and ESR. Cultures for bacteria and viruses from blood, throat, and urine were all negative. Cardiological evaluation showed a normal heart rate induced by pacemaker; neurological examination, Brainstem Auditory Evoked Potentials (BAEPs) and brain CTscan were negative. ENT evaluation revealed a moderate right sensorineural hearing loss (SNHL). Initially a diagnosis of labyrinthitis was made, and a treatment with oral prednisolone (1 mg/kg daily) was started. Nausea, nystagmus, dizziness, tinnitus and vertigo disappeared in a few days, while a persistent moderate right SNHL was confirmed in the following ENT evaluations. Ten months later, the boy referred headache, and dizziness, and was admitted in our hospital, where audiometry showed a severe right SNHL, worsened when compared to the previous examination. Neurological and cardiological evaluations were normal. Routine laboratory test and inflammatory markers were normal. The genotype study of GJB2 (connexin-26) and GJB6 (connexin-30) mutations were negative, so hereditary hearing loss was excluded. The study of NLRP3 gene mutations was normal, so Muckle-Wells syndrome was also ruled out. Autoantibodies against inner ear showed a positivity for anti-reovirus peptide antibodies, and a strong positivity for anti-peptide 12 (Cogan peptide), anti-Peptide CD148 and anti-connexin 26 antibodies. The patient was diagnosed as having autoimmune hearing loss and was started an oral prednisone (1 mg/kg/day). Headache and dizness rapidly disappeared, steroids were tapered after 3 months and hearing loss was stable.

## Conclusion

The transplacental passage of maternal antibodies, SSA/Ro and SSB/La, into the fetal circulation system induced CHB. To our knowledge, the coexistence of congenital heart block and SNHK has not been previously reported. Interestingly, the autoantibodies which are able to induce tissue damage on binding of cell-surface molecules present on the sensory epithelia of the inner ear and on endothelial cells can cross-react with anti-SSA/Ro. We are currently investigating the pathogenetic role of anti-Ro in inner ear damage.

## Disclosure of interest

None declared

